# Equine Transport and Changes in Equid Herpesvirus' Status

**DOI:** 10.3389/fvets.2018.00224

**Published:** 2018-09-25

**Authors:** Katharine E. Muscat, Barbara Padalino, Carol A. Hartley, Nino Ficorilli, Pietro Celi, Peter Knight, Sharanne Raidal, James R. Gilkerson, Gary Muscatello

**Affiliations:** ^1^School of Life and Environmental Sciences, Faculty of Science, University of Sydney, Sydney, NSW, Australia; ^2^Jockey Club College of Veterinary Medicine and Life Sciences, City University of Hong Kong, Hong Kong, Hong Kong; ^3^HKSAR- Department of Veterinary Medicine, University of Bari, Bari, Italy; ^4^Faculty of Veterinary and Agricultural Sciences, The University of Melbourne, Melbourne, VIC, Australia; ^5^DSM, Parsippany, NJ, United States; ^6^Discipline of Biomedical Science, School of Medical Sciences, University of Sydney, Sydney, NSW, Australia; ^7^School of Animal and Veterinary Sciences, Charles Stuart University, Wagga Wagga, NSW, Australia

**Keywords:** equine herpesvirus, reactivation, shedding, stress, transport

## Abstract

The risk of respiratory disease in the transported horse can increase as a consequence of immunosuppression and stress associated primarily with opportunistic bacterial proliferation and viral reactivation. This study examines the ecology of equid herpesviruses (EHV) in these horses, exploring reactivation and changes in infection and shedding associated with transport, and any potential contributions to transport-related respiratory disease. Twelve horses were subjected to an 8-h road-transport event. Antibodies to EHV-1 and EHV-4 were detected by ELISA in serum collected prior to, immediately after and 2 weeks post transport. Respiratory tract endoscopy and tracheal washes were collected prior to and 5 days after transportation. Nasal swabs collected prior to, immediately after, 1 and 5 days following transport were screened for EHV-1,-2,-4,-5 using qPCR. Six horses had persistent neutrophilic airway infiltrates post transportation, indicative of subclinical respiratory disease. No horses were qPCR positive for either of the alphaherpesviruses (i.e., EHV-1/-4) nor did any seroconvert to either virus. Four out of nine horses positive for either EHV-2 or EHV-5 on qPCR prior to transport developed neutrophilic airway inflammation. Five horses showed increasingly positive readings on qPCR (i.e., reduced Cq) for EHV-2 after transportation and seven out of eleven horses positive for EHV-2 after transport shared strains of high sequence similarity with other horses in the study. One EHV-2 virus detected in one horse after transport was genetically different which may be due to reactivation. The clinical significance of EHV-2 and EHV-5 remains in question. However these results indicate that transportation may lead to increased shedding, transmission and reactivation of EHV-2 and EHV-5 but not EHV-1/-4. Unlike previous work focusing on the role of alphaherpesviruses, this research suggests that investigation of the gammaherpesviruses (i.e., EHV-2/-5) in transport-related disease should not be dismissed, particularly given that these viruses can encode suppressive immunomodulators that may affect host health.

## Introduction

Respiratory disease is a significant economic problem for the equine industry, disrupting performance and training of athletic horses and producing outbreaks of contagious illness in herds that can result in mortalities of foals and adult horses (1). The equine alphaherpesviruses, equid herpesvirus 1 (EHV-1) and EHV-4 are among the most well-known and well-characterized of the respiratory viruses to affect horse populations, notorious for producing severe clinical signs of upper respiratory tract disease in naïve horses (2). EHV-1 produces systemic infections, with strains associated with abortion and neurological disease (3). Although more prevalent, the contribution of EHV-2 and EHV-5 to disease is less defined. These viruses are predominantly found in normal horses, however, infections have been associated with both mild and severe respiratory illness and occasionally with other non-respiratory cases (4–8). Nevertheless, gammaherpesviruses have immunomodulation capacity in the host that could potentially increase susceptibility to secondary pathogens with more severe disease outcomes (9–12).

As members of the family *Herpesviridae* these viruses are characterized by their ability to establish latent infections within the host, a period of limited gene expression followed by reactivation of viral shedding and/or recrudescent manifestation of disease (13). Like other alphaherpesviruses EHV-1 and EHV-4 establish latency in neural cells, specifically within neurons in the trigeminal ganglia and in cells of the lymphoreticular system (14). Gammaherpesviruses on the other hand prefer to establish latency in cells of the immune system such as B-lymphocytes in the case of EHV-2 and EHV-5 (15–17). Reactivation of latent infections with subsequent viral shedding has been observed naturally after stress-inducing events and has also been achieved experimentally through administration of corticosteroids (18, 19).

Transport-associated respiratory disease in the horse is primarily associated with compromise of the respiratory tract mucociliary clearance system on account of the need to maintain the horse in an upright position with restricted movement of the head. This physiological restriction, allows commensal bacteria of the upper respiratory tract to migrate down to the lungs and establish pneumonia in an opportunistic fashion (20). However, immunosuppression due to stress may also be a contributing factor in equine transport-related disease. Previous research has associated increased shedding of the alphaherpesviruses with long distance transportation. Reactivation of latent herpesviruses due to transport stress has also been suggested (21). The current study is unique in its aim to use serological and nucleic acid based techniques to identify evidence of recrudescence, potential transmission and changes in shedding of EHV-1,-2,-4, and-5 in horses following a long distance transport event, and assess the potential role of these common respiratory viruses in transportation associated respiratory disease syndromes in horses. It is hypothesized that transport stress will stimulate viral shedding and reactivation of previously latent herpesvirus infections and potentially increase susceptibility of horses to respiratory disease.

## Materials and methods

### Equine population and transport event

Twelve horses between 3 and 8 years old [Standardbreds (*n* = 8) and Thoroughbreds (*n* = 4)] were purchased for the study, including mares (*n* = 5) and geldings (*n* = 7). Horses were divided into 2 groups of 6 and transported on a truck on two separate, consecutive days. The horses were transported for 8 h in basically a single loop around the Riverina region of New South Wales. The trip was punctuated by one 15-min period of rest midway through the trip, in accordance with Animal Ethics requirements (ACEC 14/037). Vaccination and transport history was unknown with the exception of the transfer of all study animals from the same owner 3 weeks prior to the experiment. Animals were mixed within a paddock over the first week of acclimatization and no animals were in estrus. All animals were anticipated to have had some traveling experience, this was supported by a lack of any evasive behavior and minimal time taken (<3 seconds) when loading each horse onto the truck (22).

Throughout the study, horses were stabled in individual boxes with at least 1 h of paddock access each day with members of the same transport group. This grouping was maintained for 5 days prior and 5 days post transportation.

### Sample collection

Serum samples in plain Vacutainer (BD, USA) were collected prior to transport, at 12-24 h and 2 weeks after transport and were stored at -20°C until required.

Nasal swabs were collected prior to loading the horses onto the truck, at unloading following the journey, and at 1 day and 5 days after transport. Both nostrils of each horse were sampled, and the swab was transferred to saline medium within a sterile 5 mL screwcap container. Aliquots of these samples were transported to the University of Melbourne laboratory and stored at -80°C.

Clinical examination was performed on each horse prior to and 5 days after transport. Horses were examined for subclinical respiratory disease through basic clinical examination of the respiratory tract complemented by endoscopy of the upper respiratory tract and tracheal wash (TW) collection as described previously (23). Cytological analysis of TW aspirates was performed using modified methods from Tee et al. (24). TW smears were stained using Wright-Giemsa stain (Hema-tek stain pack, Sigma-Aldrich Pty Ltd, Australia) and an average of four differential cell counts on 100 cells using high-power (100X) light microscopy was expressed as a percentage. Those horses with >70% neutrophilia on cytological examination of TW were classified as having subclinical respiratory disease (*n* = 6).

All procedures were approved by the Animals Care and Ethics Committee of Charles Sturt University (ACEC 14/037).

### Detection of EHV-1 and EHV-4 specific antibodies

Levels of EHV-1 and-4 antibodies present before and 2 weeks after transport were determined using an ELISA that differentiates specific antibodies using the variable amino acid regions of glycoprotein G (gG) specific to each virus. This is consistent with the standard timeframe to detect antibody responses to herpes virus infection in horses (25). Glutathione-s-transferase (GST) fusion proteins of EHV-1 gG and EHV-4 gG were tested against each serum sample in triplicate. Likewise each sample was tested against GST alone as a negative control, as described previously (26). Antibody levels were considered to be negative if the mean optical density of the 3 test wells was <0.1, questionable if between 0.1 and 0.2, and positive if more than 0.2, as has been validated in field studies (26, 27). Seroconversion in the horses was defined as an increase in optical density of more than 0.2 above the previous absorbance reading (27).

### Detection of viral shedding by quantitative PCR (qPCR)

Nucleic acid was extracted from 200 μ*L* of nasal swab sample using an automated nucleic acid extraction system (QIAxtractor, QIAGEN) and a QIAxtractor Vx kit according to the manufacturer's protocol. Quantitative PCR assays specific for EHV-1,-2,-4, and-5 were performed using the GOTaq mastermix system (Promega) according to manufacturer's instructions using GO Taq Flexi Buffer, 1.5 mM MgCl_2_, 200 μM dNTP, 0.8 μM Syto 9 (Life Technologies), 1 U GO Taq Flexi DNA polymerase and 170 nM of each of the forward and reverse primers. Primers used for detection of each of the viruses target the glycoprotein H open reading frame (EHV-1 and EHV-5), the intergenic region between ORF73 and 74 (EHV-4) or the glycoprotein B gene (EHV-2) (8). Sizes of target amplicons for EHV-1,-2,-4 and-5 were 86, 91, 167, and 87 base pairs respectively. The final 25 μL reaction volume included 2 μL of the extracted DNA template. DNA extracted from cell cultures infected with EHV-1 [strain EHV1.438/77 (28)], EHV-2 [strain EHV2.86/67 (29)], EHV-4 [strain EHV4.405/76 (28)] and EHV-5 [strain EHV5.2-141 (30)] were used as positive controls. Dilutions of nucleic acid extracted from these reference virus cultures were included on each plate along with nuclease free water as a negative control. The qPCR was performed using the Stratagene Mx3000P instrument. Thermocycling conditions were at 94°C for 15 min, then 40 cycles of 94°C for 15 sec, 60°C annealing for 30 sec and 72°C extension for 30 sec. The melting curve analysis of each amplicon was analyzed after one cycle of 95°C for 1 min, 55°C for 30 sec and 95°C for 30 sec. The fluorescence threshold value used was the default value set by the analysis software. Samples were considered positive if they had both (i) Cycle quantification (Cq) values within the linear range of detection of the dilution series of the positive controls, and (ii) a melting temperature of the amplicon within 2 degrees of the positive controls (31). To satisfy the first criteria the positive cut off value for the EHV-1,-2,-4, and-5 specific assays were determined to be 39.35, 37.4, 34.05, and 37.04 respectively. Efficiencies of the qPCR assays for EHV-1,-2,-4, and-5 were 95, 102, 98 and 98% respectively.

### Virus isolation

Virus isolation through cell culture was attempted on all nasal swab samples. Briefly, the nasal swab infused saline sample (500 μL) was diluted with 1 mL of maintenance media (Dulbecco's minimal essential medium (DMEM), 1% v/v fetal bovine serum (FBS), 10 mM 4-(2-hydroxyethyl)-1-piperazineethanesulfonic acid (HEPES), 50 μg/mL gentamicin, 5 μg/mL amphoterin B, cotrimoxazole) and filtered through a 0.45 μm syringe filter (29, 32). Vero and rabbit kidney (RK13) cell lines are permissive and therefore selective for EHV-1 in contrast to EHV-4 that only readily replicates in equine cells (33). Semi-confluent monolayers of RK13, Vero, and equine fetal kidney (EFK) cells in 12-well tissue culture plates were inoculated with 200 μL of filtrate. After 1 h adsorption at 37°C, 5% v/v CO_2_ another 1 mL of media was added and cell culture plates were returned to incubator for daily observation for any cytopathic effect (CPE). After 8 days of incubation, cell cultures were passaged into new semi-confluent monolayers according to qPCR results. Then 5 ml of media was used to inoculate a 25 cm^2^ tissue culture flask with a mixture of RK13 or EFK cell monolayers. Monolayers were examined for a further 2 days for CPE.

### EHV-2 DNA sequencing

Nasal swabs positive on qPCR for EHV-2 were further genotyped to assess possible transmission or recrudescence. Primers targeting a portion of the glycoprotein B (gB) gene, specifically nucleotides (nts) 33737–34138 of EHV-2 (GenBank accession number NC_001650), were used for conventional PCR amplification and direct sequencing of this 402 base pair (bp) region for genotyping (34, 35). Template DNA (5 μL) generated by extraction from a positive nasal swab was amplified in a 25 μL volume PCR reaction containing GO Taq Flexi Buffer, 2 mM MgCl_2_, 200 μM dNTP mix, 0.8 U GO Taq Flexi DNA polymerase (Promega), 1.24 μM of each of the forward (5′-CAGTGTCTGCCAAGTTGATA-3′) and reverse (5′-ATGGTCTCGATGTCAAACAC-3′) primers targeting the gB gene, under the following conditions; an initial denature cycle of 95°C for 5 min, 40 cycles of 95°C for 30 sec, 60°C for 30 sec, and 72°C for 40 sec, and a final extension step of 72°C for 5 min. The PCR product was examined by agarose gel electrophoresis with SYBR Green staining, products were then gel purified (QIAquick, QIAGEN), and sequenced using the forward amplification primer and big dye terminator version 3.1 chemistry (Applied Biosystems) according to manufacturer's instructions.

Amplicons unable to be sequenced directly were cloned into a pGEMT vector (Promega) according to manufacturer's directions and transformed into *E. coli* JM109 cells. Plasmids were purified from positive clones using Wizard Plus SV Miniprep kit (Promega) according to manufacturer's instructions. One to three plasmid inserts per sample were then sequenced, as described above, using the T7 primer 5′-TAATACGACTCACTATAGGG-3′.

Chromatograms were analyzed using Geneious version 6.1.2 software (Biomatters Ltd) (36). The nucleotide sequences of EHV-2 reported in this study were submitted to GenBank under the accession numbers MH351029-MH351081. Multiple nucleotide sequence alignments were performed using CLUSTAL W (35, 37). All sequences were compared to those of the EHV-2 reference strain (strain 86/67, GenBank accession number U20824.2) to determine percentage nucleotide identity. Phylogenetic relationships amongst the sequences were estimated by the neighbor-joining (NJ) method (38, 39) using PHYML version 2.2.0 (40) and the homologous gB region of an EHV-5 reference (EHV-5 2-141/67) strain (GenBank accession number KM924295) as a designated outgroup. A bootstrap value >70% represented efficient consensus support for determination of groups (35, 41).

Further phylogenetic analysis against previously published sequences of EHV-2 glycoprotein B (excluding non-functional protein data) was performed to assess variability within this portion of gB and confirm phylogenetic relationships. Only sequences with full coverage of the gB region, specifically nucleotides 1570 to 1971 (numbered according to complete coding sequence of gB of EHV-2 strain ATCC, GenBank accession no. HQ247755) were used [*n* = 26] which consisted of 2 genome sequences (including EHV-2 reference strain), 10 complete and 14 partial coding sequences of EHV-2 gB. Information on all sequences used for this analysis including GenBank accession number, country where virus was isolated and date collected (if known) can be found in Supplementary Material (Table S1).

### Data analysis

Cycle quantification (Cq) data (number of amplification cycles before fluorescence crosses threshold) from qPCR reactions were analyzed using Genstat software 18th edition (VSN International, Hemel Hampstead, UK). Data sets displayed normal distribution upon visual inspection of Q-Q (quantile-quantile) plots. A two-sided paired *t*-test was used for comparison of Cq values for each virus prior to and post transport (for horses positive on qPCR for a given virus pre and post transport), with a significant difference in viral load defined when *P* < 0.05. An overall test between pre and post transport was made (against the lowest Cq value or most positive) after transport. Separate comparisons were made between pre-transport Cq values and those at each time point (Unloading, 1 day post, 5 days post transport). Any significant findings were further assessed with a one sided test to confirm either an increase or decrease in viral shedding. Viral strains thought to have been reactivated from latent virus were defined as those detectable only after transport and in one horse.

Odds ratios (OR) were calculated for analysis of any association between viral infection (single or concurrent) and subclinical respiratory disease. A significant association was defined as an OR of more than 1 where the 95% confidence interval did not include 1.

## Results

### Serology

All horses were seronegative for EHV-1, however, the seroprevalence for EHV-4 was 92% (11/12). Comparison of antibody levels prior to and 2 weeks post transportation showed no instances of seroconversion (increase in optical density > 0.2) of EHV-1 or EHV-4 (Table S2).

### Virus detection and shedding

**Figure 1:**
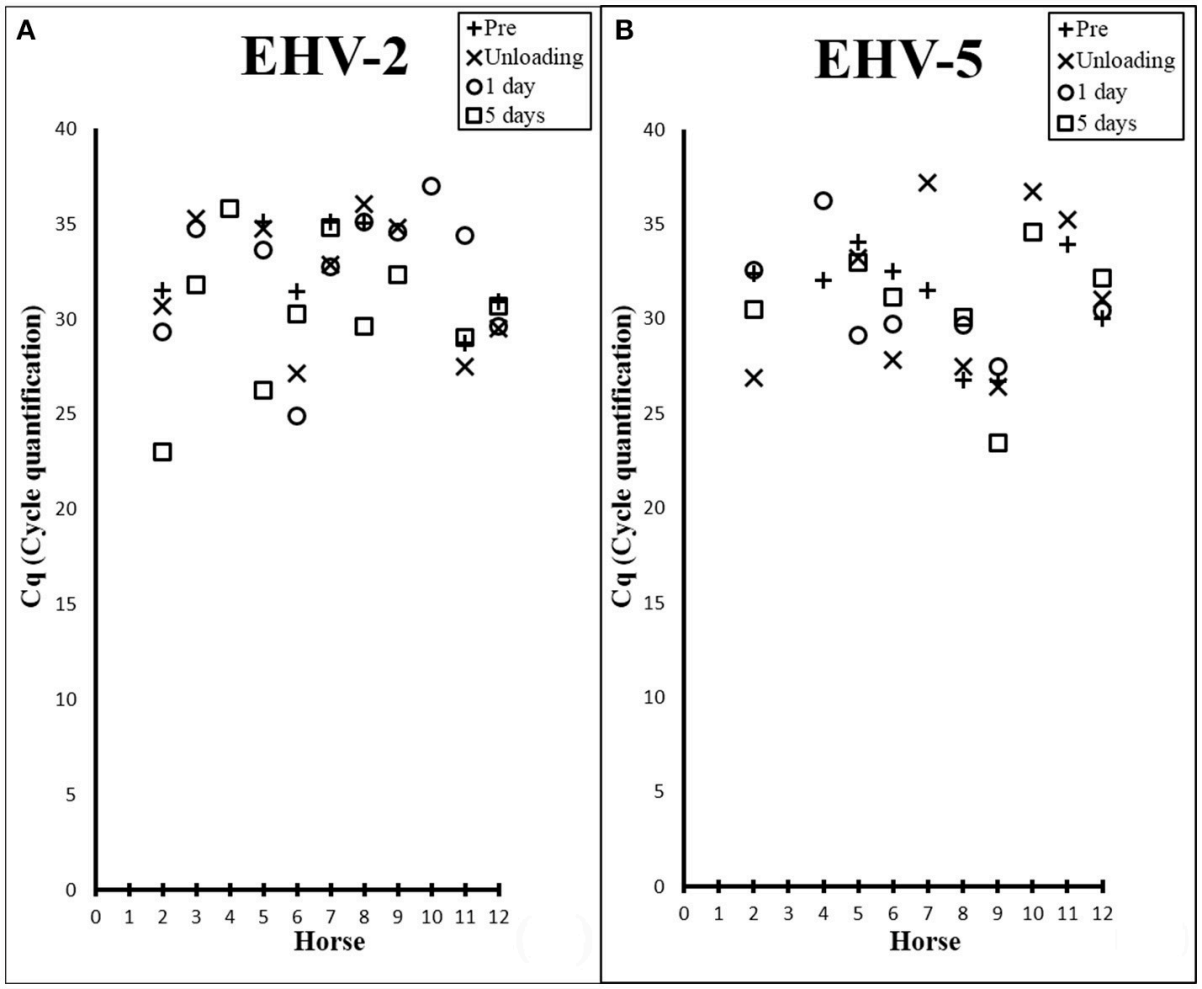
Scatter plots showing Cq values from EHV-2 **(A)** and EHV-5 **(B)** qPCR of nasal samples collected from each horse prior to transport (+), at unloading (X), 1 day post transport (○), and 5 days post transport (□).

There was a high prevalence of detection of EHV-2 (83%) and EHV-5 (75%) amongst the horses (Table 1), where melt curve analysis showed single peaks within temperature ranges of 78.95–79.8°C and 79.83–80.83°C for EHV-2 and EHV-5 samples and standards respectively. Raw Cq values for qPCR analysis are shown in Figure 1. Prevalence of EHV-2 increased over time with a total of 4 horses (Horses 3, 4, 9, 10) becoming positive after transport. The EHV-2 viral load (as indicated by Cq value) was significantly different pre-transport in comparison to lowest Cq (i.e., most positive) values after transport (*P* = 0.007). The mean difference in Cq between prior versus post was 4.9 (95% CI: 1.916, 7.898) and was confirmed to be an increase in shedding reflected by a decrease in Cq (*P* = 0.003). However, no correlation was found between a decrease in Cq and a particular time point after transport.

**Table 1:** Detection of gammaherpesviruses by qPCR in nasal swabs expressed as a percentage of study population (*n* = 12) at various time points in the transportation study.

	**Prior**	**Unloading**	**1 day post**	**5 days post**
EHV-2	58	75	83	83
EHV-5	75	75	58	58

The transportation event had no immediate effect on the prevalence of EHV-5 detection. By 1 day post transport the number of horses positive for the virus decreased by 17% [*n* = 2] (Table 1). No significant difference in EHV-5 viral load was seen in association with transport overall or at any of the various time points (Figure 1B).

All samples were negative for EHV-1 and EHV-4 by qPCR and no CPE was detected after inoculation of nasal swabs onto cell culture. The lack of CPE after 10 days in culture despite detection of EHV-2 and EHV-5 DNA by qPCR is consistent with the slow replication of these viruses in cell culture (6, 42).

A region of the gB gene of EHV-2 was sequenced for 27 of the qPCR positive nasal samples from 10 horses. No sequence could be obtained for 9 of the 36 qPCR-positive samples. Forty seven sequences were returned from these samples and had 87.6–99.3% nt identity to the reference strain. Of these, 14 sequences shared 100% similarity with at least one other sequence isolated in the study. Genomic variability of the targeted region of gB amongst EHV-2 sequences isolated from the study and those retrieved from GenBank ranged from 0–13.9% (i.e., 86.1–100% nt identity) in comparison to the homologous gB portion of the EHV-5 outgroup (EHV-5 2-141/67) that shared 75.6–81.3% sequence similarity across all EHV-2 sequences. A neighbor-joining phylogenetic tree including all EHV-2 sequences isolated from the study and those obtained from GenBank can be found in Supplementary Material (Figure S1).

**Figure 2:**
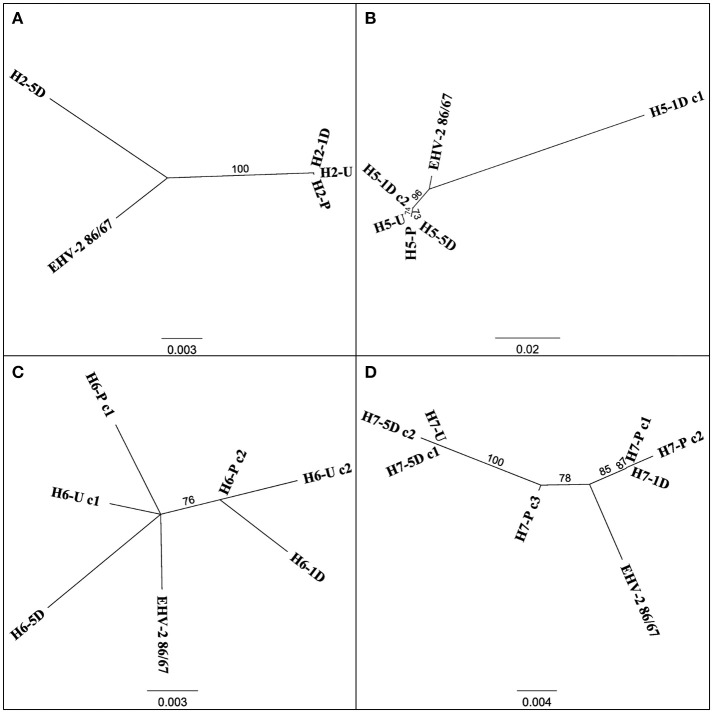
Unrooted phylogenetic trees comparing EHV-2 reference strain 86/87 and multiple strain types within individual horses 2 **(A)**, 5 **(B)**, 6 **(C)**, 7 **(D)**. Sequences are labeled according to Horse (H-), time point of sample taken (Pre Transport [-P], Unloading [-U], 1 day post transport [-1D], 5 days post transport [-5D]) and clone (c-) number if more than one sequence was isolated from each sample. Scale bars represent substitutions per site.

Multiple EHV-2 sequences were detected in seven horses over time or across replicates from the same sample. Horses 2, 5, 6, 7 appeared to be infected with multiple strains of EHV-2, nucleotide and amino acid sequence variation is summarized in Table 2. Nucleotide and amino acid alignments of sequences within each horse are provided in Supplementary Material (Data Sheets S1–S4). Figure 2 shows unrooted phylogenetic trees highlighting the genetic heterogeneity of strains within horses 2, 5, 6, and 7 and comparison against the reference strain.

**Table 2:** Genetic variation of EHV-2 sequences isolated from 4 individual horses, displayed as number of changes compared with the consensus sequence within each horse.

**Horse**	**Time point**	**Number of changes in EHV-2 gB amplicon**	
		**SNPs**	**Amino acid**
2	Pre	0	0
	Unloading	0	0
	1 day post	0	0
	5 days post	8	2
5	Pre	0	0
	Unloading	0	0
	1 day post (c1)	18	3
	1 day post (c2)	2	0
	5 days post	0	0
6	Pre (c1)	3	2
	Pre (c2)	1	0
	Unloading (c1)	4	0
	Unloading (c2)	3	0
	1 day post	3	0
	5 days post	7	0
7	Pre (c1)	4	0
	Pre (c2)	5	1
	Pre (c3)	3	0
	Unloading	6	0
	1 day post	4	0
	5 days post (c1)	5	0
	5 days post (c2)	7	1

**Figure 3:**
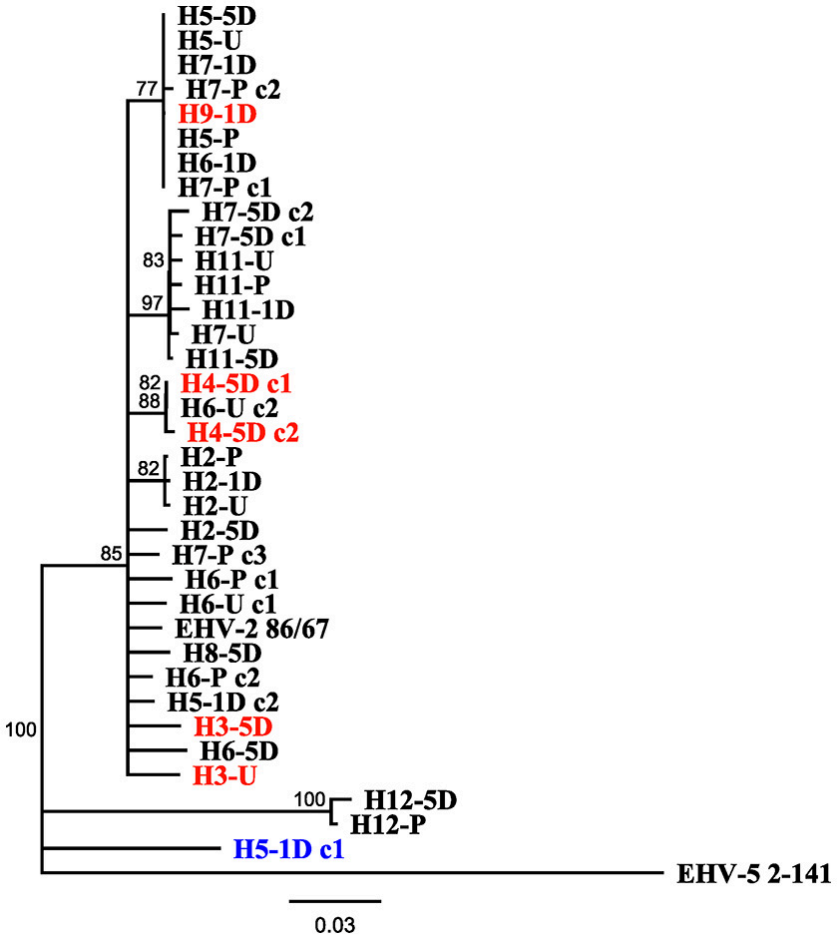
Neighbor joining phylogenetic tree illustrating relationships amongst sequences obtained from study, reference EHV-2 87/67 strain (GenBank accession number U20824.2) and outgroup EHV-5 Strain 2-141/67 (GenBank accession number KM924295). Sequences are labeled according to Horse (H-), time point of sample taken [Pre Transport [-P], Unloading [-U], 1 day post transport [-1D], 5 days post transport [-5D]] and clone (c-) number if more than one isolate was sequenced from each sample. Tip labels highlighted in red denote sequences obtained from horses positive for EHV-2 only after transport, tip labels in blue denote sequences that may have originated from reactivated virus. Scale bar represents substitutions per site.

Figure 3 displays a rooted phylogenetic tree of 34 EHV-2 sequences isolated within the study, with the inclusion of reference EHV-2 (EHV2.86/67) and EHV-5 outgroup (EHV5.2-141) strains. The majority of the sequences (n = 31) isolated in this study were clustered closely together along with the reference EHV-2 strain (Figure 3). This cluster included sequences isolated from three horses that were negative for EHV-2 prior to transport. Post transport sequences from these horses (highlighted in red in Figure 3) shared either identical or highly similar nucleotide sequences with isolates from other horses involved in the transport event. Sequences from horse 12 and a sequence from horse 5 isolated 1 day after transport were genetically distant from each other and all other isolates from the study (Figure 3).

### Subclinical disease

There were no observed signs of overt clinical respiratory disease (i.e., pneumonia) in any of the horses studied. However, half of the horses had persistent neutrophilic airway infiltrates (>70% neutrophil content) in samples collected 5 days after transportation, indicative of subclinical upper respiratory tract disease. The remaining six horses all had normal tracheal wash cytology. No association was observed between infection with either of the gammaherpesviruses prior to or post transport and incidence of subclinical upper respiratory tract disease (Table S3).

## Discussion

This study investigated the effects of transport on shedding of equid gammaherpesviruses and showed the shedding of EHV-2 increased significantly after transport, while no such pattern could be detected with EHV-5. Concurrent infections with multiple strains of EHV-2 was common within the horses and similarity of EHV-2 sequences across several horses suggested high levels of transmission. No association was found between gammaherpesvirus detection and the occurrence of subclinical transport associated respiratory disease. In addition, no evidence for reactivation of either EHV-1 or EHV-4 could be detected in association with the transport event.

Clinical signs of transport pneumonia can take 5 days or more to develop (43) hence a timeline to include the potential to observe clinical disease was included in the study of these horses' response to the transport event. Sample collection immediately and 1 day after transport allows for the detection of more immediate or acute responses to the transport event.

The detection of shedding of gammaherpesviruses in horses both before and after transport reflects the ubiquity of these viruses in horse populations (Table 1). Differences in the temporal shedding patterns between EHV-2 and EHV-5 could be consistent with a previous suggestion that reactivation of these viruses may be triggered by different biological stimuli (44). Results from a concurrent study confirmed that all horses experienced stress in relation to the transport event as shown by an increase in frequency of stress-inducing behaviors during transport and elevated cortisol levels in serum after transport (45). For horses positive for EHV-2 prior to transport, shedding significantly increased after the transport event, however, there was no association between a particular time point after transport and change in viral load. Six out of eleven horses positive for EHV-2 had highest viral load 5 days after transport according to the raw Cq data (Figure 1A) thus a significant increase in shedding was expected by this time. However, three of those six horses were negative for EHV-2 prior to transport. Since the *t*-test examines for any significant difference between 2 measurements (i.e., viral load prior and post transport) these horses could therefore not be included in the statistical analysis. The *p*-value for the comparison between viral loads prior to transport and 5 days approached significance (*P* = 0.06), hence a larger sample size may allow clarification of shedding patterns. Consideration must also be given to the possibility of concurrent infections, as up to 5 distinct EHV-2 isolates were isolated from one horse (35, 46). Similarly, genetic variation of multiple EHV-2 sequences obtained from individual horses in the current study suggests co-infection of multiple strains (Figure 2). This can complicate interpretation of the change in Cq which may not represent a change in viral load of a particular isolate.

The detection of multiple sequence types in different horses has previously been used to illustrate co-infection and re-infection of individual horses with multiple genotypes (35, 46). Using a similar approach in this study, the sequence types detected in the horses before and after transport shared 96.8% nucleotide identity and were also highly similar or identical to the sequenced region of the well characterized EHV-2 strain EHV2.86/67 (29). Horse 5 showed a uniform sequence type across all samples (pre, unloading and day 5), while the day 1 sample showed a different EHV-2 sequence with 18 SNPs and 3 amino acid changes compared to the other samples from the same horse (Table 2). This finding of a distinct virus in the series from a single horse may represent a new infection or recrudescence of a distinct sequence type over the course of the study. It is important to consider that recombination events can occur frequently between herpesviruses which in turn could impact viral sequence analysis (47). No sequence types entirely identical to that isolated from horse 5 1 day after transport were detected in the other horses (Figure 3) and therefore the origin of this virus, namely by reactivation of latent virus or re-infection from the other horses cannot be conclusively determined.

Identical or highly similar EHV-2 gB sequences isolated from nasal samples of different horses in the study (Figure 3) is consistent with shedding and horizontal transmission of EHV-2. Horses were provided with the opportunity to socialize in yards each day for 1 h allowing for exposure outside of the transport event. Phylogenetic analysis suggests that identical viral strains were detected in multiple horses that were on separate vehicles (Figure 3). Three out of four horses that became positive for EHV-2 after transport (horse numbers 3, 4, and 9) shared at least 98.1% nt identity and grouped with other isolates. As is the case with Horse 2, the notable increase in shedding over time from unloading to 5 days post transport likely represents cases of primary exposure and not reactivation of latent virus. By comparison, reactivated EHV-1 produces a relatively low viral load and shorter periods of shedding after induced reactivation from transport stress or administration of corticosteroids compared with an infection event (19, 21).

Seroprevelance of EHV-4 within the study population appears to be in accordance with previous research however the lack of seropositivity to EHV-1 in any of the horses is notable considering that exposure to EHV-1 most likely occurs early in the animal's life (27, 48). No seroconversion or nasal shedding of EHV-1/-4 after transport was detected. This reflects similar findings to that of Pusterla et al. (21) who investigated prevalence of EHV-1 nasal shedding and viremia in 302 adult horses that had undergone air transportation followed by an 8–10 day journey in a trailer. Despite exposure to a more intensive transport event only 1% (*n* = 3) of the horses shed detectable levels of EHV-1. These horses displayed no clinical signs and typically low viral copies in nasopharyngeal secretions, hence were an unlikely source of infection for other horses (49). Interestingly, shedding of EHV-4 at a sales event in South Africa was detected by qPCR within nasal secretions of 14% [*n* = 13] of 2 year old Thoroughbreds and most of these horses [*n* = 12] were seropositive to EHV-4 (50). In comparison to these horses, detection of EHV-4 nasal shedding in one horse was likely a result of a recent primary EHV-4 infection as there was a lack of a detectable antibody response. Similarly, no seroconversion was recorded. The timeframe for seroconversion to equid herpesviruses after reactivation is uncertain, hence it is possible this could have occurred after the 2 week post-transport sample point of the current study. Fecal glucocorticoid metabolite concentrations were analyzed as physiological indicators of stress and were predominantly associated with longer travel duration and days post arrival (50). The elevated levels of EHV-4 shedding in the South African study may be due to the younger age of the horses or the length of the transport in the South African study in comparison to the 8-h event and relatively older age of horses in the current study.

The absence of any relationship between viral infection and occurrence of subclinical disease is also consistent with the literature, since gammaherpesviruses are frequently detected in healthy hosts (35). Further, horse 5 which appears to have been shedding a reactivated EHV-2 virus was classed in the sub-clinically normal group. The increase in shedding of EHV-2 after transport partially supports this hypothesis, however the likelihood of transport-related reactivation of latent equine herpesviruses remains questionable from the data. Further, this study showed no detection of alphaherpesvirus shedding in the transported horses and no evidence of any clinical implications of increased shedding or recrudescence of EHV-2 after transport. However, genes have been identified in both EHV-2 and EHV-5 that produce homologues of G protein coupled receptors (GPCRs) and molecules such as interleukin (IL-) 10 that have immunomodulatory effects in other hosts (9–11, 51). Furthermore concurrent EHV-2 infection may play a role in reactivation of latent EHV-1 infections (52) and predisposing foals to *Rhodococcus equi* infection (53).

This study has shown that detection of EHV-2 and EHV-5 viral infection in horses is likely to increase with transportation due to stress-associated reactivation of latent infections or increased shedding in actively infected hosts. This shedding may also increase the opportunity for horizontal transmission in the closed airspace of the transport vehicle or subsequently after transport. This potential increase in spread of infectious agents, such as EHV, may have important implications for controlling and preventing transmission of pathogens from the transported horse, such as the time interval before such horses are integrated with others in active training or for breeding purposes. The ubiquitous nature of these viruses and their ability to concurrently infect horses makes interpretation of shedding patterns difficult. No direct association between sub-clinical respiratory disease and gammaherpesvirus infection was detected. Nevertheless to reduce the risk of infection and transmission of respiratory pathogens, stringent biosecurity measures should continue to be in place whenever horses are transported.

## Data availability statement

All relevant data are within the paper and its Supporting Information files. Sequences analyzed for this study can be found in the GenBank database under accession numbers MH351029-MH351081.

The raw data supporting the conclusions of this manuscript will be made available by the authors, without undue reservation, to any qualified researcher.

## Ethics statement

This study was carried out in accordance with the recommendations and approval of Charles Sturt University Animal Care and Ethics Committee (Project Number 14/037).

## Author contributions

GM, BP, SR, PK, and PC contributed conception of the study, design, and collection of samples. BP, KM, and NF performed laboratory and statistical analysis under supervision of CH, JG, and GM. KM wrote the manuscript with support from GM, CA, JG.

### Conflict of interest statement

The authors declare that the research was conducted in the absence of any commercial or financial relationships that could be construed as a potential conflict of interest.
