# Supplementary material for: Equine Transport and Changes in Equid Herpesvirus' Status
Source: Front Vet Sci. 2018 Sep 25;5:224. doi: 10.3389/fvets.2018.00224 (PMC6167981; doi:10.3389/fvets.2018.00224)
Supplement: Supplementary file 1 [file Table_1.DOCX]

Supplementary Material:

**Equine Transport and Changes in Equid Herpesvirus’ Status**

K Muscat^*^, B Padalino, CA Hartley, N Ficorilli, P Celi, P Knight, S Raidal, JR Gilkerson, G Muscatello

*** Correspondence:** Katharine Muscat: katharine.muscat@sydney.edu.au

# Tables

## Supplementary Table 1. Published sequences from GenBank used for EHV-2 glycoprotein B analysis

| Virus Strain | GenBank no. | Country | Year | Partial/complete cds* of gB |
| --- | --- | --- | --- | --- |
| EHV2-Bj | HQ247738 | Iceland | 2006 | Complete |
| EHV2-BB11 | HQ247739 | Iceland | 1999 | Complete |
| EHV2-gEHV-Dv | HQ247740 | Iceland | 2005 | Complete |
| EHV2-275 | HQ247750 | Switzerland | N/A | Complete |
| EHV2-276 | HQ247751 | Switzerland | N/A | Complete |
| EHV2-280 | HQ247752 | Switzerland | N/A | Complete |
| EHV2-37 | HQ247753 | Switzerland | N/A | Complete |
| EHV2-86 | HQ247754 | Switzerland | N/A | Complete |
| EHV2-ATCC | HQ247755 | Switzerland | N/A | Complete |
| EHV2-2B | HQ247756 | Switzerland | N/A | Complete |
| EHV2-G9/92 | KM924294 | UK | N/A | Genome |
| EHV2-37 | DQ486961 | USA | 2004 | Partial |
| EHV2-48 | DQ486962 | USA | 2004 | Partial |
| EHV2-34 | DQ486963 | USA | 2004 | Partial |
| EHV2-50 | DQ486964 | USA | 2004 | Partial |
| EHV2-54 | DQ486965 | USA | 2004 | Partial |
| EHV2-44 | DQ486966 | USA | 2004 | Partial |
| EHV2-52 | DQ486967 | USA | 2004 | Partial |
| EHV2-47 | DQ486968 | USA | 2004 | Partial |
| EHV2-56 | DQ486969 | USA | 2004 | Partial |
| EHV2-42 | DQ486970 | USA | 2004 | Partial |
| EHV2-40 | DQ486971 | USA | 2004 | Partial |
| EHV2-41 | DQ486972 | USA | 2004 | Partial |
| EHV2-35 | DQ486973 | USA | 2004 | Partial |
| EHV2-49 | DQ486974 | USA | 2004 | Partial |
| EHV2-86/67 | U20824 | Australia | 1967 | Full genome |
| EHV5 2-141/67 | KM924294 | Australia | 1967 | Full genome |

*cds = coding sequence

**Supplementary Table 2.** Excel file containing raw data from glycoprotein G ELISA assay detecting EHV-1/-4 antibodies in serum

**Supplementary Table 3.** Relationship between infection with EHV-2/-5 before or after transport and occurrence of subclinical disease 5 days after transport.

| **Association with disease 5 days after transport** | **Odds Ratio** |
| --- | --- |
| EHV-2 positive prior to transport | 0.5 |
| EHV-2 positive after transport | 1 |
| EHV-5 positive prior to transport | 0.4 |
| EHV-5 positive after transport | 0.2 |
| Multiple EHV-2 vs Single EHV-2 infection | 1 |
| Mixed EHV-2 infection vs no virus | 1 |

# Data Sheets

## Data Sheet S1. Nucleotide and amino acid alignment of sequences isolated from Horse 2. Single nucleotide polymorphisms and amino acid changes to the consensus sequence in Horse 2 are highlighted.

## Data Sheet S2. Nucleotide and amino acid alignment of sequences isolated from Horse 5. Single nucleotide polymorphisms and amino acid changes to the consensus sequence in Horse 5 are highlighted.

## Data Sheet S3. Nucleotide and amino acid alignment of sequences isolated from Horse 6. Single nucleotide polymorphisms and amino acid changes to the consensus sequence in Horse 6 are highlighted.

## Data Sheet S4. Nucleotide and amino acid alignment of sequences isolated from Horse 7. Single nucleotide polymorphisms and amino acid changes to the consensus sequence in Horse 7 are highlighted.

# Figures


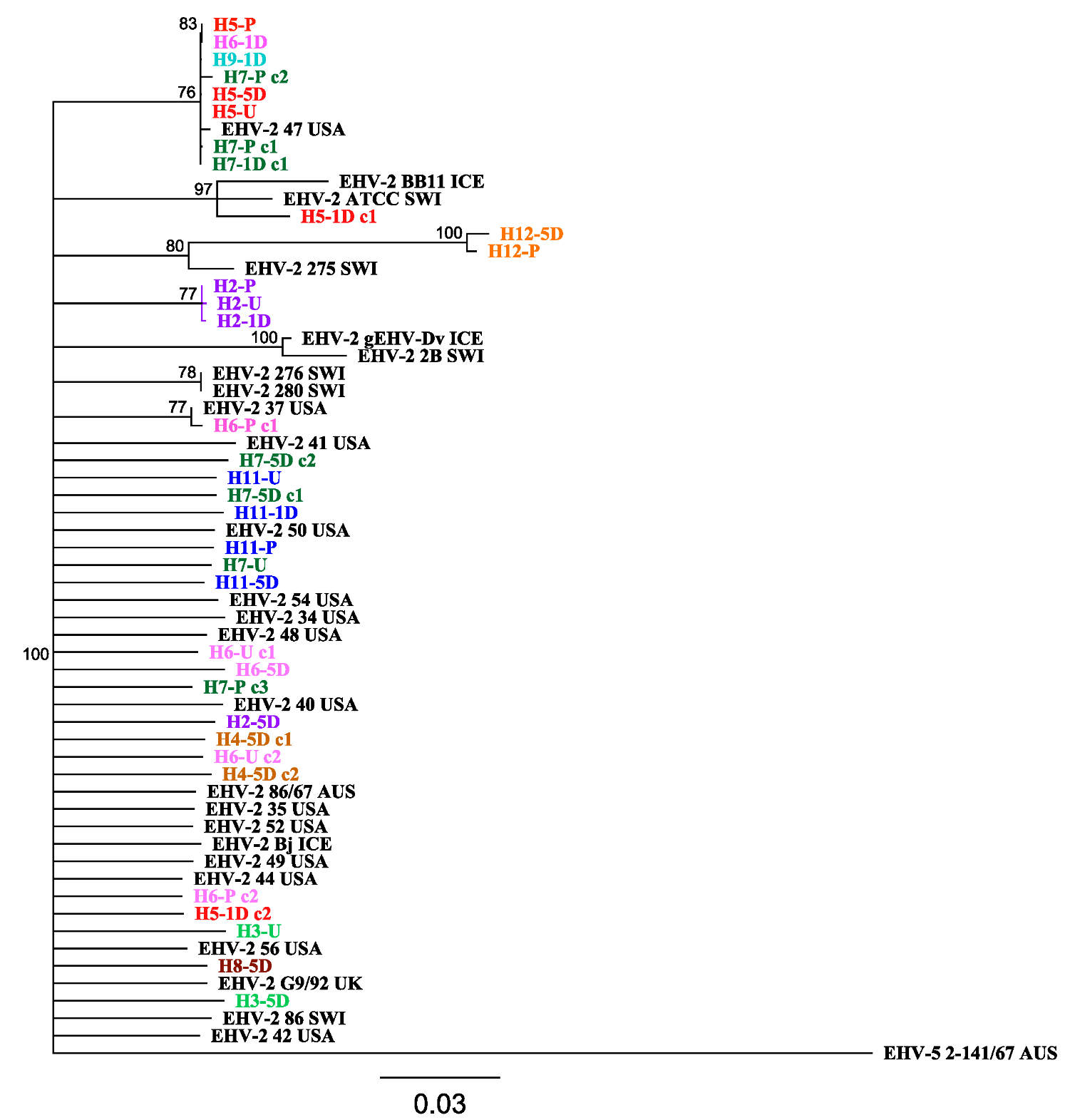


**Supplementary Figure 1.** Neighbor joining phylogenetic tree illustrating relationships amongst EHV-2 sequences obtained from study and previously published EHV-2 sequences including reference EHV-2 87/67 strain (GenBank accession number U20824.2) and outgroup EHV-5 Strain 2-141/67 (GenBank accession number KM924295). Sequences from study are labelled according to Horse (H-), time point of sample taken (Pre Transport [-P], Unloading [-U], 1 day post transport [-1D], 5 days post transport [-5D]) and clone (c-) number if more than one isolate was sequenced from each sample. Tip labels in matching colors represent sequences isolated from the same horse. GenBank sequences are denoted by EHV-2 strain name and country where strain was isolated [Australia (AUS); United Kingdom (UK); United States (USA); Iceland (ICE); Switzerland (SWI)]. Scale bar represents substitutions per site.
