# Supplementary figures and images for: Equine Transport and Changes in Equid Herpesvirus' Status
Source: Front Vet Sci. 2018 Sep 25;5:224. doi: 10.3389/fvets.2018.00224 (PMC6167981; doi:10.3389/fvets.2018.00224)

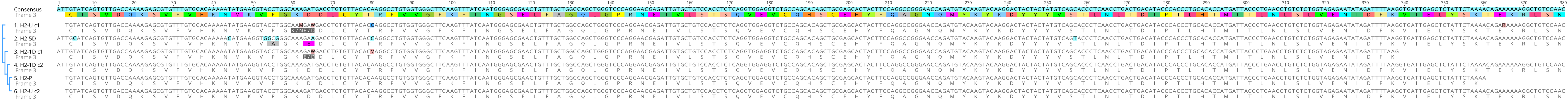

Supplement: Supplementary file 3 [file Data_Sheet_1.PDF]

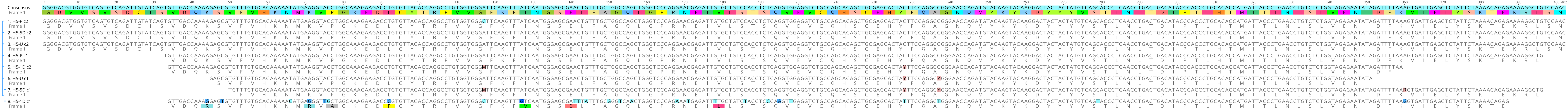

Supplement: Supplementary file 4 [file Data_Sheet_2.PDF]

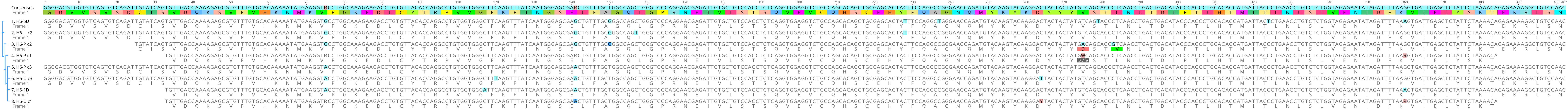

Supplement: Supplementary file 5 [file Data_Sheet_3.PDF]

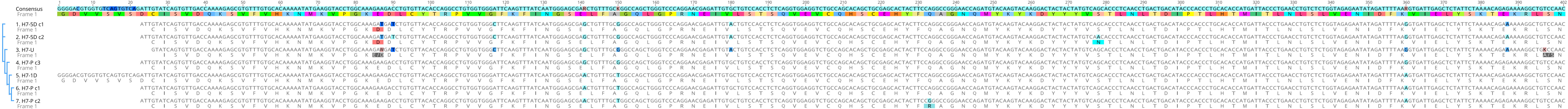

Supplement: Supplementary file 6 [file Data_Sheet_4.PDF]

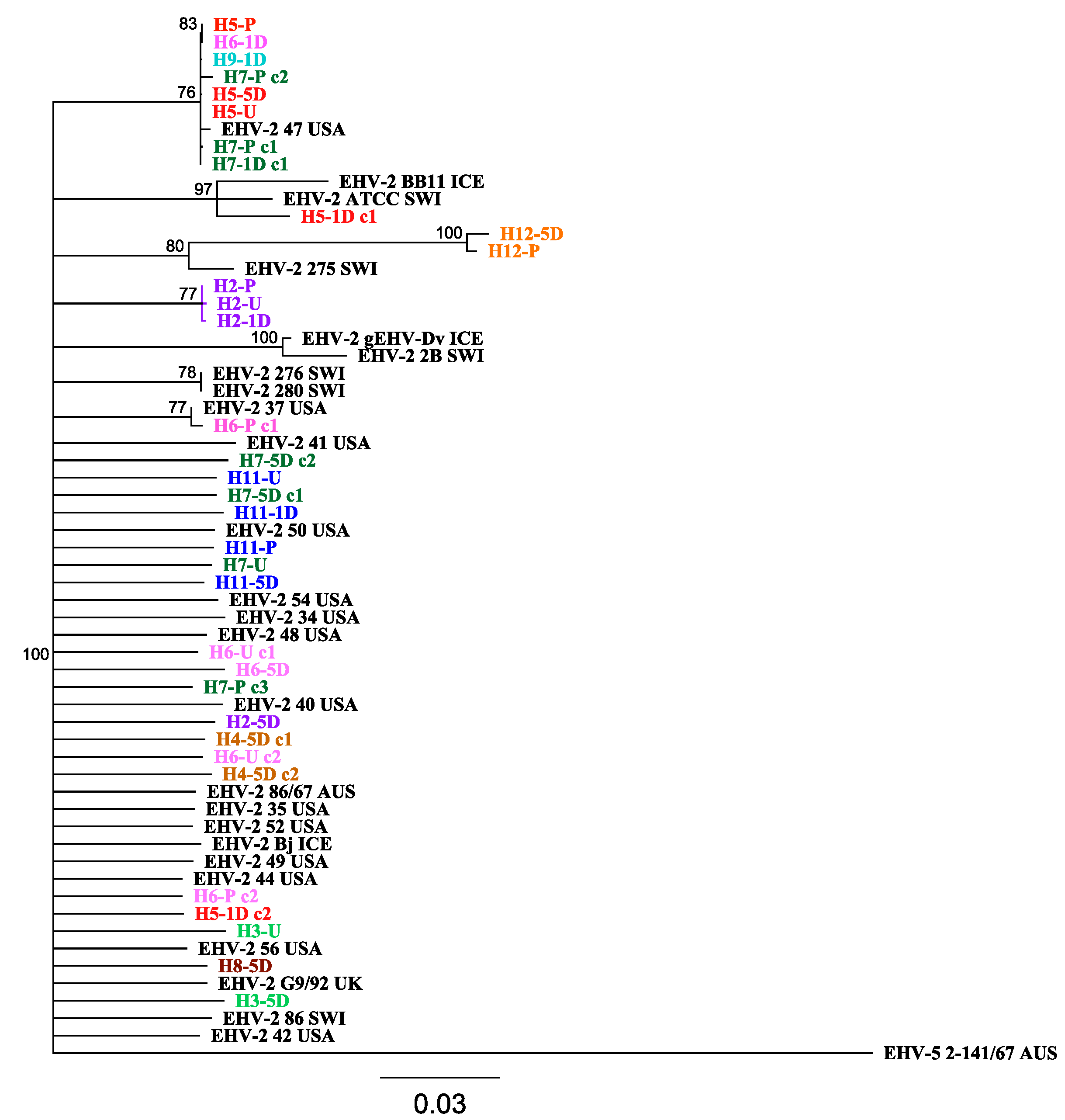

Supplement: Supplementary file 7 [file Image_1.TIF]
